# ^123^I-FP-CIT SPECT imaging in early diagnosis of dementia in patients with and without a vascular component

**DOI:** 10.3389/fnsys.2015.00099

**Published:** 2015-07-01

**Authors:** Marina Garriga, Marta Milà, Manzoor Mir, Raid Al-Baradie, Sonia Huertas, Cesar Castejon, Laura Casas, Dolors Badenes, Nuria Giménez, M. Angels Font, Jose M. Gonzalez, Maria Ysamat, Miguel Aguilar, Mark Slevin, Jerzy Krupinski

**Affiliations:** ^1^Neurology Unit, Hospital Universitari Mútua TerrassaTerrassa, Spain; ^2^Psychiatric Unit, Hospital Universitari Mútua TerrassaTerrassa, Spain; ^3^College of Applied Medical Sciences, Majmaah University AlmajmaahAl Majmaah, Saudi Arabia; ^4^Research Unit, Research Foundation Mútua Terrassa, Universitat de BarcelonaBarcelona, Spain; ^5^Cetir-Grup Medic, CTD, Hospital Universitari Mútua de TerrassaTerrassa, Spain; ^6^School of Healthcare Science, Manchester Metropolitan UniversityManchester, UK

**Keywords:** dementia, mild cognitive impairment, Framingham risk score, ^123^I-FP-CIT SPECT

## Abstract

Alzheimer’s disease (AD) and vascular dementia (VaD) are the most common cause of dementia. Cerebral ischemia is a major risk factor for development of dementia. ^123^I-FP-CIT SPECT (DaTScan) is a complementary tool in the differential diagnoses of patients with incomplete or uncertain Parkinsonism. Additional application of DaTScan enables the categorization of Parkinsonian disease with dementia (PDD), and its differentiation from pure AD, and may further contribute to change the therapeutic decision. The aim of this study was to analyze the vascular contribution towards dementia and mild cognitive impairment (MCI). We evaluated the utility of DaTScan for the early diagnosis of dementia in patients with and without a clinical vascular component, and the association between neuropsychological function, vascular component and dopaminergic function on DaTScan. One-hundred and five patients with MCI or the initial phases of dementia were studied prospectively. We developed an initial assessment using neurologic examination, blood tests, cognitive function tests, structural neuroimaging and DaTScan. The vascular component was later quantified in two ways: clinically, according to the Framingham Risk Score (FRS) and by structural neuroimaging using Wahlund Scale Total Score (WSTS). Early diagnosis of dementia was associated with an abnormal DaTScan. A significant association was found between a high WSTS and an abnormal DaTScan (*p* < 0.01). Mixed AD was the group with the highest vascular component, followed by the VaD group, while MCI and pure AD showed similar WSTS. No significant associations were found between neuropsychological impairment and DaTScan independently of associated vascular component. DaTScan seems to be a good tool to discriminate, in a first clinical assessment, patients with MCI from those with established dementia. There was bigger general vascular affectation observable in MRI or CT in patients with abnormal dopaminergic uptake seen on DaTScan.

## Introduction

Dementia is a chronic brain syndrome which affects 35.6 million people worldwide and this number is expected to triple by 2050 (115.4 million). Thus, it has an enormous impact on health care provision currently costing the world more than US$ 604 billion per year (Wimo et al., 2013).

The most frequent cause of dementia in the elderly is Alzheimer’s disease (AD), followed by vascular dementia (VaD), Parkinson disease with dementia (PDD) and Lewy Body dementia (LBD). Together these account for over 90% of cases of dementia in the elderly (Albanese et al., 2007).

**Figure 1:**
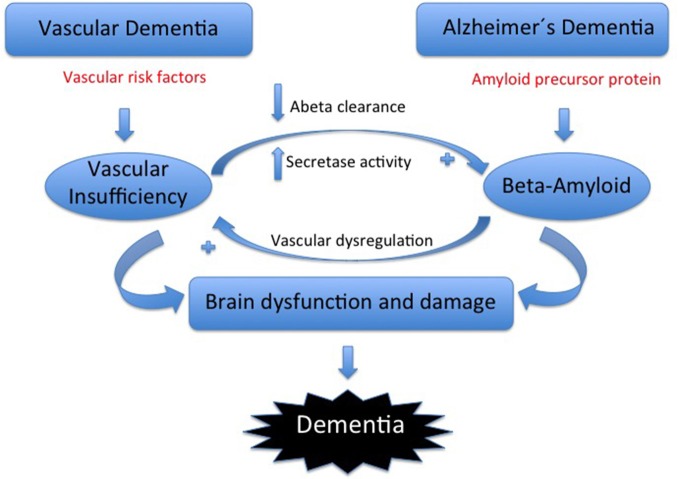
**In VaD, cerebrovascular risk factors induce neurovascular dysfunction leading to brain dysfunction and damage**. In Alzheimer’s disease (AD), cleavage of amyloid precursor protein by by β- and γ-secretases leads to Aβ accumulation, which also causes brain dysfunction and damage. Although individually these pathways are capable of inducing cognitive impairment, their interaction enhances their pathogenic effects. Thus, Aβ induces vascular dysregulation and aggravates vascular insufficiency, thereby enhancing the brain dysfunction and damage associated with vascular risk factors. In addition, the hypoxia-ischemia resulting from the vascular insufficiency increases Aβ cleavage from APP and reduces Aβ clearance through the cerebral vasculature, promoting Aβ accumulation and the attendant deleterious effects on the brain (Iadecola, 2010).

Although they have distinct features, the different types of dementia overlap clinically and pathologically, especially in the early stages and particularly in regards to the contribution of brain ischemia (Iadecola and Gorelick, 2003). Increasing evidence demonstrates that ischemia is not only an additive cause of brain damage in all types of dementia but also contributes specifically to the underlying disease processes (Niwa et al., 2002; Iadecola and Gorelick, 2003) Many cardiovascular risk factors (CVRF), such as hypertension, diabetes, dyslipidemia, and smoking increase the risk of AD, suggesting a vascular contribution to the etiology of AD. Ischemia upregulates and deregulates the entire amyloidogenic cascade. Within the framework of the neurovascular unit, vascular dysfunction may reduce the clearance of β-amyloid (Aβ) via the blood brain barrier or indirectly increase Aβ deposition. Amyloid deposition is considered the pivotal event in the AD pathological cascade, but whether the accumulation is accelerated by CVRF remains unclear (Breteler, 2000; Purnell et al., 2009; Figure 1). The evidence is stronger for VaD and AD, but there is a lack of research into the potential contribution of ischemia to LBD or PDD.

Other studies showed that most CVRF also increase the risk for AD (Tatemichi et al., 1994; Tanimukai et al., 1998; Kalaria, 2012). In large epidemiological studies, the prevalence of AD is reported to increase with the severity of atherosclerosis, the presence of atrial fibrillation, hypertension, diabetes mellitus, hyperinsulinemia and insulin resistance, and a previous stroke (Skoog et al., 1996; Leibson et al., 1997; Ott et al., 1997). Antihypertensive treatments can prevent stroke and also the risk of dementia (Sörös et al., 2013). Further, abnormalities in the cerebral white matter thought to be ischemic in nature are more common in AD than controls, and its severity correlates with mild cognitive impairment (MCI) progression to AD (Scheltens et al., 1992; Eckerström et al., 2011).

The occurrence of both AD pathology and vascular brain injury is very common, especially amongst the oldest of old and can be classified as mixed AD. Although epidemiologic studies report that vascular risk factors for arteriosclerosis increase the risk of incident AD, both autopsy, MRI and amyloid positron emission tomography (PET) studies indicate that AD and vascular lesions contribute additively, but independently, to the risk of dementia. The literature confirms the malignancy of AD and highlights the adverse effects of microinfarcts on cognitive function. For the clinical diagnosis of mixed AD, the presence of AD can be recognized by neuropsychological profile, structural imaging, cerebrospinal fluid biomarkers, and glucose PET and amyloid PET imaging. The diagnosis of mixed AD, however, still hinges predominantly on the structural MRI findings (Chui and Ramirez-Gomez, 2015). Studies on neuroimaging have reported that over 70% of patients consulting for neurodegenerative disease have abnormal MRI findings suggestive of a vascular component (Mills et al., 2007). White matter hyperintensities (WMHs) on brain MRI reflect cardiovascular risk profiles, and greater WMH volume is associated with cerebral hypometabolism and cognitive decline, particularly in tests of executive function (DeCarli et al., 1995; Jeerakathil et al., 2004; Carmichael et al., 2010). These structural and cognitive changes were associated with CVRF. The Framingham Stroke Risk Profile score, a composite risk score of CVRF that measures 10-year probability of stroke, was negatively associated with total cerebral brain volume and positively related to prevalent WMHs. The association between multiple CVRF was examined by a number of studies using the Framingham Stroke Risk Profile. High Framingham scores correlate with poor cognitive performance (Wolf et al., 1991; Elias et al., 2004; Jeerakathil et al., 2004). These results confirm that ischemia is a major risk factor for dementia. In fact, 10% of stroke patients develop dementia shortly after their first stroke and more than a third develop dementia after recurrent strokes. Subjects not developing dementia as a direct consequence of a stroke have a 10-fold increase in the risk of dementia over the next 5 years (Pendlebury and Rothwell, 2009).

Neuroimaging techniques play a major role as diagnostic instruments (Visser et al., 2012). In particular, emission tomography (single photon emission computed tomography, SPECT and PET) provides a unique tool to investigate functional and neurochemical changes, both in those with established dementia and at risk of subsequent cognitive decline (de Souza et al., 2012; Herholz, 2012).

AD is characterized by bilateral temporoparietal hypoperfusion on SPECT and hypometabolism on PET, which may precede the onset of clinical dementia. Similar changes can be demonstrated in those with MCI and in those genetically at risk of developing AD (Brun and Englund, 1986; Vanitallie, 2013). In turn, VaD is related to multiple, asymmetric, perfusion deficits secondary to brain multi infarction (Chen et al., 2013).

In contrast, in LBD medial parietal and occipital perfusion deficits are seen together with mainly pre-synaptic dopaminergic changes, most commonly a reduction in the striatal pre-synaptic dopamine transporter (DAT) which can be visualized using appropriate tracers [e.g., ^123^I-N-3-fluoropropyl-2beta-carbomethoxy-3beta-4-iodophenyl tropane (^123^I-FP-CIT SPECT); McKeith et al., 2005; Cummings et al., 2011].

Thus, advanced functional imaging has a promising potential in supporting dementia diagnosis. Indeed, imaging of the DAT defines integrity of the dopaminergic system, and hence its degeneration *in vivo* (Varrone and Halldin, 2010). Its main clinical application is in patients with mild, incomplete, or uncertain Parkinsonism (Weng et al., 2004; Tolosa et al., 2007; Eshuis et al., 2006). That is, striatal uptake correlating with disease severity, in particular bradykinesia and rigidity. Also, it is a useful tool in the monitoring of progression in clinical trials of potential neuroprotective drugs and in differentiating juvenile-onset Parkinson’s disease (abnormal ^123^I-FP-CIT SPECT) from dopa-responsive dystonia (normal ^123^I-FP-CIT SPECT; Marshall and Grosset, 2003).

Dopamine loss is seen even in the earliest clinical presentations of true Parkinsonism; a normal scan suggests an alternative diagnosis such as essential tremor, drug-induced Parkinsonism, or psychogenic Parkinsonism (Cummings et al., 2011). Congruence between working clinical diagnosis and DAT imaging increases over time in favor of baseline ^123^I-FP-CIT SPECT imaging results. Additional applications, especially accurate when combining with other neuroimaging tools such as FDG-PET, characterize dementia with Parkinsonian features (Cummings et al., 2011; Garibotto et al., 2013). Furthermore, it is possible to differentiate AD from LBD [normal tracer uptake in AD and abnormal in dementia with Lewy bodies (DLB) with sensitivity and specificity of 78−88% and 94−100%, respectively; McKeith et al., 2005].

Regarding the vascular component involved in dementia, the role of nuclear medicine imaging in the diagnosis of vascular Parkinsonism (VP) or VaD has been addressed by only a few studies up to now and with non-definitive results. Some of these suggest normal ^123^I-FP-CIT SPECT (Gerschlager et al., 2002; Lorberboym et al., 2004; García Vicente et al., 2005) in VP while others suggest reduced ^123^I-FP-CIT binding as well as a lower mean asymmetry index than Parkinson’s disease (Zijlmans et al., 2007). In general more studies reported higher percentage of normal FP-CIT SPECT in VP patients (Bouwmans et al., 2013).

The aim of this study was to analyze the vascular contribution towards dementia and MCI. We evaluated the utility of FP-CIT SPECT for the early diagnosis of dementia in patients with and without a clinical vascular component, and the association between neuropsychological function, vascular component and dopaminergic function on FP-CIT SPECT.

## Materials and Methods

### Subjects

The study population included 105 patients. They were recruited prospectively from the outpatient Dementia and Stroke Unit. We included patients with clinical diagnosis of MCI (*n* = 50), probable dementia (AD and VaD, *n* = 37), and Parkinson disease with dementia (PDD and DLB, *n* = 18; Table 1).

**Table 1:** **Distribution of different diagnoses in our study group**.

	N	Age	Sex M:F
Mild cognitive impairment (MCI)	50	72 ± 8	26:24
Alzheimer’s disease (AD)	31	76 ± 7	12:19
Parkinson disease with dementia (PDD)	12	77 ± 6	10:2
Vascular dementia (VaD)	6	73 ± 9	3:3
Lewy body dementia (LBD)	6	73 ± 11	3:3

*Data are presented as mean (±SD) or number*.

Dementia was diagnosed according to the *Diagnostic and Statistical Manual of Mental Disorders*, fourth edition (DSM-IV), and AD based on the criteria of the *National Institute of Neurological and Communicative Disorders and Stroke* and the *Alzheimer’s Disease and Related Disorders Association* for definite, probable, or possible. Independently, two trained neurologists made the diagnosis and the final diagnosis was established by consensus. Patients with hemispheric vascular accident or severe isolated aphasia were not included. Also, patients with prior cranial trauma, severe heart, kidney or liver disease, cancer or infection signs were excluded from our study.

### Baseline Assessment

#### Demographic and Clinical Variables and Cardiovascular Risk Profile

Demographic data included age and gender. We also recorded medical history including data such as CVRF and psychiatric disorders. CVRF were extracted from the medical history: hypertension, dyslipidemia, Diabetes Mellitus, smoke habit, family history of dementia or cardiovascular disease, presence of peripheral vascular disease (PVD), coronary artery disease (CAD) or prior stroke. Cardiovascular risk score was calculated using the recently developed Framingham General Cardiovascular Disease Risk from the Framingham Heart Study (Wolf et al., 1991; D’Agostino et al., 2008). Framingham Risk Score (FRS) was estimated in all patients according to age, gender, total cholesterol levels, HDL levels, systolic blood pressure, hypertension treatment, smoking habit, diabetic status, and vascular disease (CAD, PVD, stroke). Those patients presenting moderate or high FRS were categorized as having a clinical vascular component, while all the rest were considered as not having it.

Data were provided by the patient and/or caregiver or extracted from medical reports if available.

#### Physical Examination

Neurological clinical examination was performed. Assessments were undertaken according to the local practice. The Mini-Mental State Examination (MMSE) was administered as a cognitive screening test. The effect of cognitive impairment on global functioning was measured with the Clinical Dementia Rating Scale (CDR) and the Global Deterioration Scale (GDS). Instrumental activities of daily living (IADL) and ADL functions were measured with the Blessed Dementia Rating Scale (BDRS), the Bayer ADL or self-rating Bayer ADL scale.

Motor sub-scale of the Unified Parkinson’s Disease Rating (UPDRS- III) was used as a measure to help in the description of the extrapyramidal symptoms (EPS). Neuropsychiatric symptoms were measured with the Neuropsychiatric Inventory (NPI) and depression rates on the elderly were measured with the Yesavage scale.

#### Neuropsychological Battery

A standardized protocol was administered by a trained neuropsychologist. We selected a short version of the Barcelona test. The domains tested were orientation, attention, immediate and differed memory, language, praxis, gnosis, executive functions and working memory. Neuropsychologists were also asked to give a GDS of the cognitive impairment evaluated.

#### Complementary Physical Examinations

General blood tests were realized including lipid profile. According to our laboratory criteria, lipid abnormal profile was defined as cholesterol levels higher than 200 mg/dl, high density lipoprotein cholesterol (HDL) lower than 56 mg/dL if men and lower than 65 mg/dL if women, low density lipoprotein (LDL) cholesterol higher than 100 mg/dl and triglycerides higher than 190 mg/dL.

#### Neuroimaging Study

In addition to the clinical vascular component defined using FRS, the vascular contribution in each group of patients was also quantified from WMHs on anatomic neuroimaging.

##### MRI and CT imaging

MRI was used preferentially, if not available CT was used. MRI was performed on a Symphony MR A-35 1.5 T scanner and processed using MR 2004 A Syngo software. CT imaging was performed on Siemens Sensation Somaton E scanner and processed using the software CT 2007 –S 16 C. Axial CT or T2 weighted MRI images were used for the quantification of the vascular component using a validated score according to Wahlund scale (from 0 to 3; Wahlund et al., 2001). The Wahlund scale was used in the analyses of WMHs in five cerebral areas in both hemispheres: frontal, parieto-occipital, temporal lobes, infratentorial area and basal ganglia. WMHs on MRI were defined as ill-defined hyperintensities >5 mm on T2 images, and on CT as ill-defined and moderately hypodense areas of >5 mm. Lacunes were described as well-defined areas of >2 mm with attenuation (on CT) or signal characteristics (on MRI) the same as cerebrospinal fluid. If lesions with these characteristics were <2 mm, they were considered perivascular spaces. Changes in the basal ganglia were rated in the same way and considered WMHs even if located in the gray matter nuclei, which contains a small amount of white matter. The Total Score of the Wahlund Scale (WSTS) was finally quantified as the mean value of the five cerebral areas analyzed.

##### Molecular imaging of dopaminergic activity by ^123^I-FP-CIT SPECT

SPECT images were acquired on a 2 headed gamma camera hybrid SPECT-TC Infinia HW4 General Electronic with a high resolution collimator. Images were acquired 3 h after a single intravenous injection of 5 mCi (111–185 MBq) of the radiotracer ^123^I Ioflupane. Subjects underwent standard thyroid blocking with potassium iodide oral administration (120 mg) 1–4 h before and 12–24 h after the radiotracer injection. Images were assessed using a dichotomous division of normal (0) *vs*. lower uptake (1).

### Statistical Analyses

Demographic parameters were expressed as absolute values and percentages for the qualitative variables and by mean and standard deviation for the quantitative variables.

In bivariate analysis, qualitative variables were compared using the χ^2^ test or Fisher exact probability Test, when appropriated. To complete the comparison between qualitative and quantitative variables Student’s *t*-test was carried out. For the mean comparisons ANOVA test was used. Finally, Pearson correlation coefficients were used to examine the relationship between quantitative variables. A two-sided *P* value of <0.05 was used to assess statistical significance. For significant variables, 95% confidence intervals (CI) were established.

Statistical analyses were performed using the Statistical Package for the Social Sciences SPSS 17.0 (SPSS Inc., Chicago, IL, USA).

### Ethical Considerations

This study was done in accordance with the Review Board and Ethics Committee of our center. Written informed consent was always obtained from patients.

## Results

### Subject Demographics

Subject’s demographic characteristics, CVRF, results of neurological, neuropsychological and neuroimaging examination frequencies of MCI group compared to the dementia group are shown in Table 2. In Table 3 the same features are described as N(%) for each dementia type and also MCI.

**Table 2:** **Subject’s demographic characteristics, distributions of CVRF, results of neurological, neuropsychological and neuroimaging examination in MCI group compared to the dementia group**.

*N* (%)	MCI	Dementia
Sex M:F	26 (52):24 (48)	29 (53):26 (47)
Age	72 ± 8	75 ± 8
Vascular component	14 (29)	14 (26)
EPS	0 (0)	17 (31)
		
Hypertension	29 (62)	31 (66)
Dyslipidemia	16 (34)	17 (36)
Diabetes mellitus	12 (26)	15 (32)
Smoking or ex-smoking	10 (21)	10 (21)
PVD	2 (5)	3 (7)
CAD	4 (10)	6 (14)
Prior stroke	15 (37)	11 (26)
		
Total cholesterol	194 ± 40	190 ± 47
HDL cholesterol	50 ± 18	51 ± 21
LDL cholesterol	108 ± 63	103 ± 55
Triglycerides^♯^	137 ± 100	171 ± 59
		
HACHINSKI	4 ± 3	4 ± 3
GDS *fast*	3 ± 1	4 ± 1
MMSE^♯^	25 ± 4	21 ± 5
UPDRS-III *motorsub-scale*^♯^	9 ± 9	37 ± 15*
		
WSTS^♯^	0.5 ± 0.4	0.7 ± 0.6
^123^I-FP-CIT SPECT decrease uptake^♯^	7 (21)	14 (45)

Numbers and % are presented for each group. ^♯^p < 0.05; *This refers only to PDD group.

**Table 3:** **Subject’s demographic characteristics, CVRF, results of neurological, neuropsychological and neuroimaging examination in different dementia subtypes and in MCI patients**.

*N* (%)	AD	VaD	PDD	LBD	MCI
Vascular component	7 (23)	5 (83)	2 (17)	0	14 (28)
EPS	0	0	12 (100)	6 (100)	0 (0)

Hypertension	18 (67)	4 (80)	8 (80)	1 (17)	29 (62)
Dyslipidaemia	9 (33)	2 (40)	4 (40)	2 (33)	16 (34)
Diabetes mellitus	6 (22)	2 (40)	4 (40)	3 (50)	12 (26)
Smoking or ex-smoker	6 (22)	0	2 (20)	2 (33)	10 (21)
PVD	1 (4)	1 (20)	1 (11)	0	2 (5)
CAD	3 (13)	1 (20)	2 (22)	0	4 (10)
Prior stroke	5 (21)	3 (60)	2 (22)	1 (17)	15 (37)

Total cholesterol	205 ± 49	165 ± 39	172 ± 34	186 ± 62	194 ± 4
HDL cholesterol	56 ± 17	66 ± 30	36 ± 22	47 ± 14	50 ± 18
LDL cholesterol	136 ± 48	83 ± 22	82 ± 56	74	108 ± 63
Triglycerides	144 ± 65	84 ± 31	97 ± 46	117 ± 68	137 ± 100

HACHINSKI	4 ± 3	6 ± 5	5 ± 3	2 ± 2	4 ± 4
GDS *fast*	4 ± 1	NA	4 ± 1	NA	3 ± 1
MMSE	22 ± 5	22 ± 6	19 ± 3	19 ± 6	25 ± 4
UPDRS-III *motor sub-scale*	NA	NA	38 ± 17	33 ± 4	9 ± 9

WSTS	0.6 ± 0.5	0.7 ± 0.6	0.8 ± 0.5	0.3 ± 0.4	0.5 ± 0.4
^123^I-FP-CIT SPECT lower uptake	3 (23)	0 (0)	7 (78)	4 (80)	7 (21)

*Numbers and percentage are presented*.

### Vascular History and Risk Factors

Within clinically diagnosed AD, 7 out of 31 patients had vascular lesions on imaging and were classified as mixed AD. However, none of the CVRF studied was associated with dementia. Similar frequencies of hypertension were seen in MCI and dementia (62 *vs*. 66%, respectively). The same smoking habit frequency was found in MCI compared to dementia (21%). Zero percent smokers were found in the VaD group and the highest frequency existed in DLB (40% smokers).

HDL levels were normal for all the different subtypes of dementia except DLB and PDD, which presented with lower levels (36 ± 22 mg/dL and 47 ± 13 mg/dL, respectively). Elevated LDL levels were found in AD (136 ± 48 mg/dL). Overall dementia presented with general abnormal triglycerides levels (171 ± 59 mg/dL).

Regarding the clinical component according to FRS, no significant association was found with dementia compared to MCI (frequencies of 64% and 63%, respectively). When studying the different types of cognitive impairment separately, PDD showed the highest frequency (92%) followed by VaD (67%), MCI (63%), DLB (60%) and AD (53.6%).

Related to the presence of PVD this showed the highest frequency within the VaD group (20%) and no significant differences were observed between MCI (5%) and overall dementia (7%). The dementia group presented with higher frequency (14 *vs*. 10% in MCI) of CAD, and similar frequencies were seen in AD, VaD, PDD (13, 20 and 22% respectively). MCI patients had more prior strokes than dementia patients (37 *vs*. 26%). When ischemic lesions were examined according to Hachinski ischemic scale, VaD had the highest score (6 ± 5 total score).

### Parkinsonism Features

The presence of extrapyramidal signs (EPS) was measured with UPDRS III (motor subscale) test. In our sample, 17 patients presented with EPS, corresponding to all patients with PDD and DLB. UPDRS III scores were higher in PDD than in MCI (mean values of 37 *vs*. 9 in a 0–108 scale).

### Cognitive Test Function and Neuropsychological Battery

Our population of probable dementia patients did not show significant differences regarding MMSE scores among dementia subtypes (mean MMSE score for probable dementia and MCI was 21 and 25, respectively).

A significant association was found between dementia and the global neuropsychological function, measured as a summary of the different affected areas (*p* = 0.025).

When we analyzed each neuropsychological area separately, patients with overall dementia (regardless of dementia subtype) had a higher frequency of affectation of the orientation domain compared to those with MCI (35% in MCI and 58% in dementia, *p* < 0.05). Speech was also more affected in dementia (16% in MCI and 39% in dementia, *p* < 0.05) and gnosis alterations was not found in MCI (0%) while 13% of patients with dementia presented with gnosis alterations (*p* < 0.05). No significant differences were found in relation to the other tested functions (memory, working memory, attention, praxis).

#### Association Between Vascular Component and WSTS

We used the WSTS to quantify the vascular lesions observed either in MRI or CT and then studied their association with the qualitatively established clinical vascular component.

An association was found between the presenting clinical vascular component according to FRS and WSTS calculated using MRI and CT images. Patients presenting with a clinical vascular component showed a higher mean WSTS (mean WSTS 0.7 *vs*. 0.4, *p* < 0.05 respectively).

We also found significant association regarding frontal, basal ganglia, infratentorial and Wahlund Scores with vascular component according to FRS (*p* < 0.05).

#### Association Between WSTS and Clinical Diagnoses: Mixed AD has Higher WSTS than Other Dementia Types or MCI

**Figure 2:**
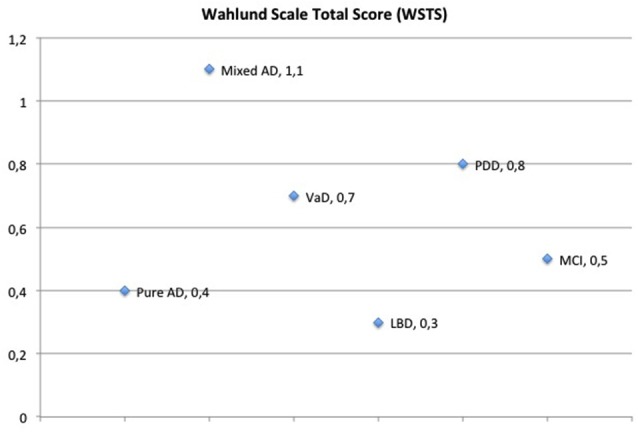
**Mean values of wahlund scale total score (WSTS) measured on MRI/CT for each patient group**. AD patients with clinical vascular component (mixed AD) have the highest score, followed by VaD and PDD. DLB showed the lowest score, while mild cognitive impairment (MCI) and pure AD presented with comparable values.

One of our aims was to study the contribution of the clinical vascular component towards brain damage and dementia. Hence, after seeing association between clinical vascular component according to FRS and WSTS, we wanted to look at the association between having a high WSTS and the clinical diagnoses of either dementia or MCI. Although not being statistically significant, the highest mean WSTS was found in the PDD group of patients. Moreover, when AD patients with clinical vascular component (mixed AD, 7/31 patients) were separated from the pure AD they appeared to have the highest WSTS. In contrary, pure AD showed a lower WSTS, even lower than that observed in MCI (Figure 2).

Association between area-specific WSTS and clinical diagnosis was only found in infratentorial WSTS, which is linked to PDD (*p* < 0.05).

#### Association Between Clinical Diagnoses and Uptake in ^123^I-FP-CIT SPECT

**Figure 3:**
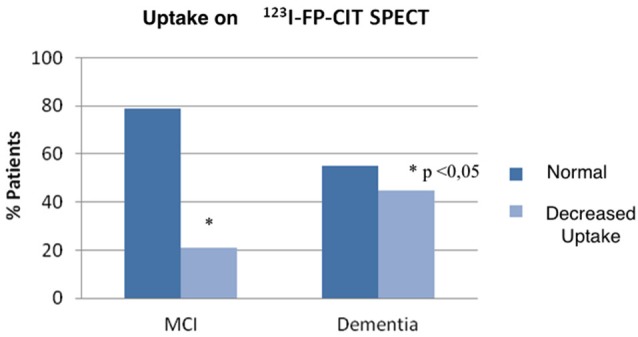
**Dopaminergic uptake distribution observed on ^123^I-FP-CIT SPECT for MCI and dementia group**.

^123^I-FP-CIT SPECT was available in 33 MCI and 31 dementia patients (13/31 AD, 4/6 VaD, 9/12 PDD, 5/6 LBD, respectively), a total of 61% of studied population. A significantly higher frequency of decreased uptake in ^123^I-FP-CIT SPECT was found in patients with dementia compared to MCI (21% patients with decreased uptake in MCI *vs*. 45% patients in dementia group, *p* < 0.05; Figure 3; Table 2). As expected, patients with Parkinsonism (DLB and PDD) showed the highest frequency of decreased dopaminergic uptake (80% in DLB and 78% in PDD; Table 3).

#### Association Between WSTS and Dopaminergic Uptake in ^123^I-FP-CIT SPECT

**Figure 4:**
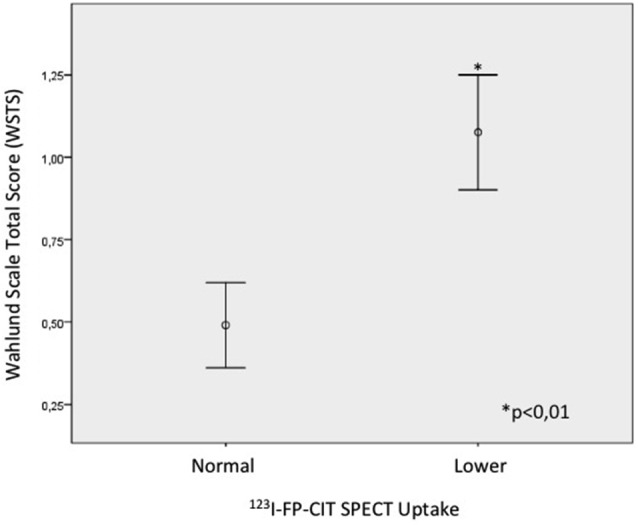
**Association between WSTS and abnormal dopaminergic uptake in ^123^I-FP-CIT SPECT (*p* < 0.01)**.

**Figure 5:**
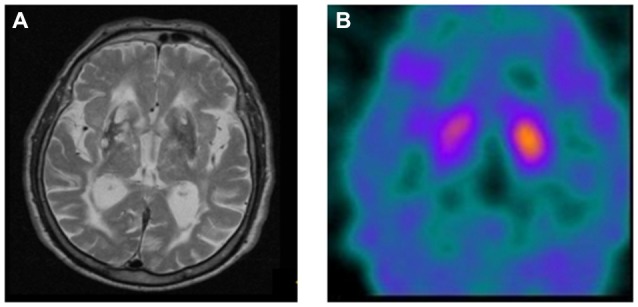
**MRI and ^123^I-FP-CIT SPECT Images from a representative patient to show relation existing between abnormal MRI and uptake on ^123^I-FP-CIT SPECT. (A)** MRI T2-potentiated showing diffuse cerebral atrophy with increased size and depth of cerebral grooves and ventricular supratentorial enlargement. Multiple lacunar chronic infarctions, some of those were hemeorrhagic in the lenticular and head of caudate nuclei. Chronic periventricular ischemia leucoencephalopaty and focal hyperintensities in parietal frontal and temporal bilateral white matter WSTS = 1.6. **(B)** ^123^I-FP-CIT SPECT from the same subject showing detectable decreased uptake in right putamen.

A significant association was found between dopaminergic uptake seen on ^123^I-FP-CIT SPECT and the vascular component quantified using WSTS (*p* < 0.01; Figure 4). Thus, patients presenting higher WSTS more frequently showed an abnormal ^123^I-FP-CIT SPECT image (*p* < 0.01; Figure 5).

Nevertheless, no significant association was found between an abnormal ^123^I-FP CIT SPECT image and the vascular component according to FRS.

#### No Association Between Dopaminergic Uptake ^123^I-FP-CIT SPECT nor WSTS and Neuropsychological Function

In relation to the association between dopaminergic uptake observed in ^123^I-FP-CIT SPECT and the neuropsychological function examined by an ordinary test battery. No significant results have been obtained when studying each neusopsychological function separately, and neither with the overall performance on neuropsycological tests. No relationship was found between neuropsychological function and the clinical vascular component measured using WSTS.

## Discussion

In this study we aimed to analyze the contribution of vascular component (assessed clinically and on neuroimaging) to clinical diagnoses of the common types of dementia and MCI. We tested the potential application of ^123^I-CIT-SPECT imaging in dementia, particularly when the vascular component is present. We looked at the association between having vascular component and presenting an abnormal ^123^I-CIT-SPECT. Finally, we studied the association between neuropsychological function and vascular component or abnormal ^123^I-CIT-SPECT. A better understanding of the association between these variables could help providing new tools for a more accurate and early diagnoses of common types of dementia.

Our results showed an association between clinical vascular component according to FRS and quantification of the vascular component using WSTS on MRI and CT images. These results are in line with what would be expected and confirm the reliability of the quantification of WMHs lesions using Wahlund scale in our population (Wahlund et al., 2001). Further, it confirms that it was a well selected population. The association between WMHs for each brain area separately was significantly different in frontal, basal ganglia and infratentorial areas. However, no association was found within the temporal area, which actually would be the most likely, considering that most frequent types of AD-like dementia are characterized by temporo-parietal alterations (Fischer et al., 1990; Jabłoński et al., 2011; Marchesi, 2011).

It was observed in previous studies that vascular lesions contribute to cognitive decline. It was suggested that cerebrovascular lesions induce amyloid deposition perhaps accelerating the process of dementia (Garcia-Alloza et al., 2011; Pluta et al., 2013). Although, we did not find a significant association between the different clinical diagnoses of cognitive impairment and WSTS, we have observed a notable frequency of the vascular component on neuroimaging within the AD group. The later group probably was formed by mixed AD population. These findings confirmed the fact that vascular accident history contributes to the etiology and/or progression of dementia. Patients with mixed AD and PDD had a high WSTS, even higher than in patients with VaD. Although not being statistically significant, these results are in line with the association between AD or PDD and WMHs. No conclusions can be drawn related to overall dementia and MCI WSTS.

In relation to the particular areas affected by ischemic lesions and each diagnoses, we only found a significant association relating to the infratentorial region in PDD. This could be explained by the fact that in our PDD group there was a high frequency of VP, as this group presented with a high mean of WSTS (0.8 ± 0.5) with 92% frequency of clinical vascular component according to FRS. Further, the non-association of VaD and any specific brain region could be due to a diffuse pattern of this type of dementia, unusual low FRS of this group and/or the few number of patients in this group.

Furthermore, we evaluated the potential utility of ^123^I-FP-CIT SPECT as a diagnostic tool to discriminate dementia from MCI, and differentiate among different dementia types, emphasizing on dementia with a vascular component. Our study showed that ^123^I-FP-CIT SPECT is a useful tool to discriminate MCI from overall dementia subtypes. This is a promising observation, since it is a major clinical priority to be able to more accurately confirm dementia diagnoses and predict MCI progression. However, in our study we could not demonstrate the utility of ^123^I-FP-CIT SPECT to discriminate among different dementia types.

As expected, most of patients with PDD and all but one of those with LBD showed an abnormal ^123^I-FP-CIT SPECT. Nevertheless, three patients with PD did not show an abnormal ^123^I-FP-CIT SPECT, suggestive of possible iatrogenic Parkinsonism. We hypothesized that patients with VaD could show an abnormal ^123^I-FP-CIT SPECT if ischemic lesions affected directly or indirectly the dopaminergic control of cortical-striatal circuits. This was not seen in our study probably because of the small number of patients with VaD (*n* = 6), none of which showed abnormal ^123^I-FP-CIT SPECT.

Once we had demonstrated the utility of ^123^I-FP-CIT SPECT to differentiate dementia *vs*. MCI, we then studied the association between abnormal ^123^I-FP-CIT SPECT and presence of the vascular component clinically assessed with the FRS and quantified on neuroimaging using WSTS. An association between the vascular component according to FRS and abnormal ^123^I-FP-CIT SPECT was not found, but the association between dopaminergic function and WSTS was statistically significant. Thus, our results showed that ^123^I-FP-CIT SPECT discriminates between vascular risk factors load and vascular component quantified using WSTS and that neuroimaging of vascular lesions is indeed important. Wahlund scale criteria thus being more accurate in analysing the grade of vascular component observable than classical FRS. It is interesting that WSTS, as a global brain WMHs score, rather than basal ganglia sub-score, is associated with an abnormal ^123^I-FP-CIT SPECT imaging. This suggests that this technique can reflect vascular alterations other than those affecting basal ganglia. The association observed between abnormal ^123^I-FP-CIT SPECT and the vascular component quantified by WSTS could be justified because dementia patients have a significantly higher frequency of abnormal ^123^I-FP-CIT SPECT, in particular, the PDD group seems to be the responsible for this finding. PDD and LBD had the highest frequency of abnormal ^123^I-FP-CIT SPECT and PDD showed the second highest WSTS after mixed AD. Moreover, AD showed a high vascular contribution (high mean WSTS) and the frequency of abnormal ^123^I-FP-CIT SPECT was higher (23%) than expected according to previous reports that confirm the utility of ^123^I-FP-CIT SPECT in the differentiation of PDD/LBD from AD (O’Brien et al., 2009; Cummings et al., 2011). Some studies reported that approximately 5–10% of ^123^I-FP-CIT SPECT in patients with clinical dementia showed intermediate scans. Thus, abnormal images within AD group may represent mixed LBD/AD disease (Kemp and Holmes, 2007).

In conclusion, our study supports the utility of ^123^I-FP-CIT SPECT to detect abnormal dopaminergic function in those patients that showed a high WSTS corresponding to a high grade of generalized ischemic brain lesions. This decrease uptake could be due to the loss of dopaminergic innervation in the striatum as a consequence of ischemia or the alteration of dopaminergic system because of abnormal cell functionality, characteristic of oxidative stress that could precede cell death in dementia or MCI (Kim et al., 2012).

There is growing evidence demonstrating that the vascular component has an active role in dementia mechanisms and its progress and is probably involved in most dementia subtypes which share similar physiopathological features (Iadecola and Gorelick, 2003). Therefore, it is not surprising that an abnormal ^123^I-FP-CIT SPECT is associated with cerebrovascular lesions related to dementia, but cannot differentiate among dementia subtypes that have an important vascular component, such as mixed AD, VaD or VP.

It is known that ^123^I-FP-CIT SPECT is useful to discriminate AD from PDD/LBD, and hence to distinguish patients in whom dopaminergic therapy may be beneficial (Varrone and Halldin, 2010; Cummings et al., 2011). Therefore, an abnormal scan suggests underlying neurodegeneration, supportive of a diagnosis of Parkinson’s disease, or atypical parkinsonism, LBD and even VP if the nigrostriatal system is affected. Conversely, a normal dopaminergic imaging supports an alternative diagnosis such as AD, essential tremor or iatrogenic parkinsonism. In our study, we demonstrated that ^123^I-FP-CIT SPECT could be also useful to confirm diagnosis of dementia among those patients with MCI.

Recent studies have demonstrated a 6–8% decline of DAT per decade (Erixon-Lindroth et al., 2005), suggesting DAT binding is a powerful mediator of age-related cognitive changes. These findings should be taken under consideration when designing and interpreting *in vivo* imaging studies.

Finally, this study did not find a significant association between neuropsychological function and abnormal ^123^I-FP-CIT SPECT nor high WSTS. This suggests that neither measurement of vascular component score nor the functionality of dopaminergic system can detect degree of cognitive decline in our sample.

### Limitations

It is important to note that patients with VaD in our sample had lower FRS scores than expected; the distribution of CVRF was low and they did not appear to have the highest WSTS-being lower than those observed in AD, where cholesterol levels seemed to be a risk factor to develop AD. This fact could be due to the small number of patients in our sample, especially in the VaD group. Another limitation of our study was the use of clinical diagnosis as the gold standard, which may not always be accurate.

Further studies are needed to better elucidate the potential role of dopaminergic transporter imaging on detecting vascular brain damage and its association with clinical dementia.

## Conclusion

In agreement with previous reports ^123^I-FP-CIT SPECT imaging is abnormal in patients with extrapyramidal signs (PDD and DLB). Interestingly, around 20% of patients with pure AD or MCI had abnormal SPECT. The new finding is that on initial visit ^123^I-FP-CIT SPECT can differentiate severity in terms of classification between MCI and dementia, thus helping establish more accurately initial diagnosis and maybe providing a useful tool to discriminate MCI from overall dementia at clinical practice. Furthermore, in our sample, the vascular contribution is shown to be relevant and present among AD and PDD patients, in line with previously reported data and in addition, there is a good correlation between uptake on ^123^I-FP-CIT SPECT and ischemic score on MRI or CT.

## Author Contributions

All authors contributed to work. Marta Milà and Marina Garriga participated in patients selection, screening and follow up. Manzoor Mir and Raid Al-Baradie helped with analysis of data, Sonia Huertas, Cesar Castejon, Miguel Aguilar, Jerzy Krupinski evaluated patients, helped with manuscript, Laura Casas and Dolors Badenes performed neuropsychological tests. Marta Milà, Raid Al-Baradie, Manzoor Mir analyzed molecular images, Marta Milà and Marina Garriga quantified MRIs. All authors contributed to the final version of the manuscript.

## Conflict of Interest Statement

The authors declare that the research was conducted in the absence of any commercial or financial relationships that could be construed as a potential conflict of interest.
